# Association between time-to-antibiotics and clinical outcomes in neutropenic fever after hematopoietic stem cell transplant

**DOI:** 10.1007/s00520-026-11141-8

**Published:** 2026-10-28

**Authors:** Samuel L. Windham, Christopher A. Guidry, Anthony D. Sung, Marin H. Kollef, Steven Q. Simpson

**Affiliations:** 1 Division of Infectious Diseases, University of Kansas Medical Center, Kansas City, KS USA; 2 Acute Care Surgery Division, University of Kansas Medical Center, Kansas City, KS USA; 3 Division of Hematologic Malignancies and Cellular Therapeutics, University of Kansas Medical Center, Kansas City, KS USA; 4 Division of Pulmonary and Critical Care Medicine, Washington University School of Medicine, St. Louis, MO USA; 5 Present Address: Division of Pulmonary and Critical Care Medicine, University of Kansas Medical Center, Kansas City, KS USA

**Keywords:** Neutropenic fever, Hematopoietic stem cell transplant, Time-to-antibiotics, Bacteremia

## Abstract

**Background:**

Guidelines recommend empiric antibiotics within one hour for neutropenic fever, extrapolated primarily from sepsis literature. Whether modest variation in time-to-antibiotics (TTA) affects outcomes in antibiotic-naïve hematopoietic stem cell transplant (HSCT) recipients remains unclear.

**Methods:**

We conducted a retrospective cohort study of neutropenic fever episodes occurring within 30 days of HSCT at a single academic center (2018–2022). We included antibiotic-naïve episodes (no intravenous antibiotics in the 24 h preceding fever) with timestamp-validated TTA. Primary outcome was clinical deterioration (new vasopressor requirement ≥ 6 h, mechanical ventilation, or 90-day mortality). Secondary analyses examined bacteremia and blood culture yield.

**Results:**

Among 224 antibiotic-naïve episodes, 17 (7.6%) experienced clinical deterioration. Median TTA was 1.2 h (IQR 0.8–2.2), with 86 (38.4%) receiving antibiotics within one hour. In the primary categorical analysis, TTA was not associated with deterioration (≤ 1 h vs > 1 h, adjusted OR 1.18, 95% CI 0.37–3.78, P = .78); a per-hour continuous parameterization was similarly null (adjusted OR 1.03, 95% CI 0.85–1.24, P = .80). Recognized pathogen bacteremia occurred in 59 episodes (26.3%) and was not associated with 90-day mortality (adjusted OR 1.98, 95% CI 0.50–7.90, P = .33); a sensitivity analysis using any-positive cultures (69/224 [30.8%]) yielded similar findings (OR 1.15, 95% CI 0.31–4.28, P = .84). Of 11 deaths, 10 (91%) occurred without preceding acute deterioration. Pre-antibiotic and post-antibiotic cultures had similar recognized pathogen positivity (27.1% vs 23.5%; adjusted OR 1.03, 95% CI 0.28–3.85, P = .97).

**Conclusion:**

In antibiotic-naïve adult HSCT recipients with neutropenic fever, TTA was not associated with deterioration and bacteremia was not associated with mortality. Per-hour TTA effects were bounded below the minimum detectable effect size, challenging the extrapolation of the 1-h antibiotic rule from septic shock cohorts to this population. Multicenter validation is needed.

**Supplementary information:**

The online version contains supplementary material available at https://doi.org/10.1007/s00520-026-11141-8.

## Introduction

Neutropenic fever is among the most common and potentially life-threatening complications following hematopoietic stem cell transplantation, occurring in the majority of patients during the pre-engraftment period.[1–3] The 2010 IDSA febrile neutropenia guideline emphasizes prompt empiric antibiotic therapy but does not specify a precise hourly threshold for adult inpatient febrile neutropenia; the explicit 1-h standard originates in the 2018 ASCO-IDSA outpatient management update, framed for emergency department and ambulatory presentation.1,[4] This 1-h threshold has subsequently been operationalized as the inpatient standard at major adult cancer centers, including the MD Anderson Cancer Center, Memorial Sloan Kettering, Cleveland Clinic, and Thomas Jefferson.[5–8] However, this recommendation is derived primarily from the sepsis and critical care literature, where delays in antibiotic administration have been associated with increased mortality in patients presenting with septic shock.[9–11] Whether neutropenic fever represents a fundamentally different disease process, in which the 1-h standard derived from septic shock does not apply, has not been directly tested in adult HSCT recipients.

The existing evidence base for time-to-antibiotics in febrile neutropenia is mixed. A 2020 systematic review of 13 studies found inconsistent associations between antibiotic timing and outcomes, with a pooled odds ratio for mortality of 0.78 (95% CI 0.16–3.69) reflecting substantial heterogeneity across study populations and methods.[12] Studies demonstrating benefit from rapid antibiotics have often emerged from settings with prolonged baseline treatment times, where delays may reflect systemic deficiencies or late recognition.[13] In contrast, studies from centers with already-rapid antibiotic delivery have generally failed to demonstrate outcome differences within the first few hours.[14–17] A recent target trial emulation in pediatric febrile neutropenia similarly found no association between antibiotic timing and serious adverse events after propensity score adjustment.[18].

The pathophysiology of fever in early post-transplant neutropenia may differ from sepsis presenting to the emergency department: conditioning regimen toxicity causes mucosal barrier injury leading to transient translocation of enteric organisms that may trigger fever and bacteremia without the fulminant trajectory of community-acquired sepsis.[19–23] Whether timing thresholds derived from septic shock cohorts should govern management of this pathophysiologically distinct syndrome remains an open question.

To address this gap, we analyzed timestamp-validated neutropenic fever episodes within the first 30 days following HSCT at a single academic medical center. Our primary aim was to assess the association between time-to-antibiotics and clinical deterioration, defined as new vasopressor requirement, mechanical ventilation, or death. Two secondary analyses tested assumptions implicit in the 1-h standard. First, we examined the association between bacteremia and mortality, since the link between treatable infection and outcome is the presumed mechanism by which faster antibiotics improve survival. Second, we characterized the relationship between antibiotic administration and blood culture yield, since pursuit of a 1-h target can shift culture collection into the post-antibiotic window and ultimately affect culture yield.

## Methods

### Study design and setting

We conducted a retrospective cohort study of adult hematopoietic stem cell transplant (HSCT) recipients who developed neutropenic fever within 30 days of transplantation at a single academic medical center between January 2018 and December 2022. This study was approved by the Washington University Institutional Review Board (Protocol #202,212,067) with a waiver of informed consent.

#### Antimicrobial prophylaxis

Antibacterial, antifungal, antiviral, and *Pneumocystis jirovecii* prophylaxis were administered per institutional protocol, with selection varying by transplant type, conditioning regimen, and graft-versus-host disease risk. Antifungal options included micafungin, fluconazole, posaconazole, and voriconazole. Episode-level prophylaxis exposure is summarized in Table 1.

**Table 1:** Baseline Characteristics

Characteristic	Antibiotic-Naïve Cohort (N = 224)
Age, median (IQR), y	60.0 (50.0–66.0)
Female sex, No. (%)	96 (42.9)
Race, No. (%)	
White	195 (87.1)
Black	24 (10.7)
Other/Unknown	5 (2.2)
Hispanic ethnicity, No. (%)	4 (1.8)
Underlying disease, No. (%)	
Myeloma	94 (42.0)
AML	39 (17.4)
Myelodysplastic syndrome	1 (0.4)
Lymphoma	45 (20.1)
ALL	11 (4.9)
Otherᵇ	34 (15.2)
Allogeneic transplant, No. (%)	68 (30.4)
Days from HSCT to NF, median (IQR)	6.7 (3.9–8.6)
ANC nadir ≤ 500 cells/µL within 24 h of fever, No. (%)	210 (93.8)
SOFA score, median (IQR)	4.0 (3.0–5.0)
Age-adjusted Charlson, median (IQR)	1 (0–4)
Blood culture obtained, No. (%)	219 (97.8)
Positive blood culture, No. (%)	69 (30.8)
Time to empiric GN antibiotic, median (IQR), h	1.2 (0.8–2.2)
Antibiotics administered ≤ 1 h, No. (%)	86 (38.4)
Clinical deteriorationᵃ, No. (%)	17 (7.6)
Vasopressor requirement	7 (3.1)
Mechanical ventilation	1 (0.4)
90-day mortality	11 (4.9)
Antimicrobial prophylaxis, No. (%)	
Antiviral (acyclovir/valacyclovir)	213 (95.1)
Antifungal (any)	86 (38.4)
Antibacterial (fluoroquinolone)	12 (5.4)

^a^ Defined as vasopressor requirement ≥ 6 h, mechanical ventilation, or 90-day mortality. ^b^ Other includes aplastic anemia, rare hematologic malignancies, and cases where primary diagnosis was not specified in the transplant database

## Definitions

### Neutropenic fever

Body temperature was measured by oral electronic thermometry per institutional nursing protocol, with values documented in the structured electronic health record flowsheet. Fever was defined as a single temperature ≥ 38.3 °C or temperature ≥ 38.0 °C sustained for at least 2 h. We used the 2-h sustained criterion, more conservative than the 1-h threshold in some guidelines, to reduce inclusion of transient elevations unlikely to reflect true infection. Importantly, at this institution antibiotics were usually started with the first temperature ≥ 38.0 °C, so the 2-h sustained criterion acts as a means to classify actual febrile events, not the start of antibiotic timing. Neutropenia was defined as an absolute neutrophil count < 1500 cells/µL, corresponding to Grade 1 neutropenia in the Common Terminology Criteria for Adverse Events version 5.0.[24] This threshold reflects institution-wide clinical practice. A neutropenic fever (NF) episode required fever and neutropenia occurring within ± 24 h of each other. Fever events occurring within 72 h were merged into a single episode, with the first qualifying fever defining episode onset. This mirrors clinical practice and literature on duration of therapy for culture-negative neutropenic fever.[25].

### Antibiotic exposure

We identified all intravenous antibiotic administrations using medication administration records with validated timestamps. Empiric gram-negative (GN) coverage was defined as receipt of cefepime, piperacillin-tazobactam, meropenem, ceftazidime, or aztreonam. Time-to-antibiotics (TTA) was calculated as the interval from the first qualifying fever timestamp (a single temperature ≥ 38.3 °C, or the first ≥ 38.0 °C reading of a fever that met the 2-h sustained criterion) to first empiric GN antibiotic administration. Antibiotic-naïve episodes were defined as those with no intravenous antibiotics and no empiric GN antibiotics in the 24 h preceding fever onset.

### Blood cultures

Blood culture sets were linked to NF episodes if specimen collection occurred within ± 24 h of fever onset. We applied two bacteremia classifications. The recognized-pathogen definition (excluding coagulase-negative staphylococci as sole isolates and excluding fungal isolates; retaining viridans group streptococci, *Leptotrichia*, *Granulicatella*, and other commensal organisms as recognized pathogens given their established pathogenicity in post-HSCT neutropenia) was the primary classification, used for the bacteremia-mortality analysis, the culture-timing analyses, and the transplant-stratified analyses. The any-positive definition (recovery of any bacterial isolate) was used for the pathogen distribution and as a sensitivity for the bacteremia-mortality analysis. For 5 episodes whose susceptibility testing had been performed at an external reference laboratory or had not been completed, organism identity was reconciled from primary microbiology records.

### Clinical deterioration

The composite deterioration outcome was defined as new vasopressor requirement for ≥ 6 h, new mechanical ventilation, or discharge to hospice/death within 90 days. Vasopressor requirement and mechanical ventilation were assessed within 72 h of fever onset; mortality was assessed within 90 days of episode onset. Components were not mutually exclusive.

### Severity assessment

Sequential Organ Failure Assessment (SOFA) scores were calculated at NF onset using the worst vital signs in the 72 h preceding and 6 h following fever onset and the worst laboratory values in the 24 h preceding fever. The age-adjusted Charlson comorbidity index was calculated using pre-transplant diagnoses, excluding the index hematologic malignancy.

### Source of infection

Non-bacteremic infectious sources were extracted from the structured EHR within the 7-day fever window: CT-confirmed pneumonia (manual review of chest CT radiology reports), urinary tract infection (urine culture ≥ 105 CFU/mL of a recognized urinary pathogen), tracheal aspirate bacterial culture growth, *Clostridioides difficile* infection (stool toxin-positive), respiratory viral co-infection (respiratory viral panel positivity), and cytomegalovirus viremia (CMV PCR positivity in serum). Source-specific denominators are restricted to episodes with the corresponding diagnostic testing performed.

### Mucositis

Oral mucositis grade per CTCAE v5.0 was extracted from the structured EHR record. The per-episode grade was the maximum recorded from 48 h before through 24 h after fever onset.

### Study cohorts

The full cohort comprised all NF episodes occurring 0–30 days after HSCT (N = 492 episodes in 437 patients) and was used for descriptive analyses, microbiological characterization, and as a sensitivity for the bacteremia–mortality analysis. The antibiotic-naïve cohort comprised episodes in which empiric GN antibiotics were administered within 12 h of fever onset following no intravenous antibiotic exposure in the preceding 24 h (N = 224); this was the primary cohort for both the time-to-antibiotics and bacteremia–mortality analyses. For culture-timing analyses, we examined antibiotic-naïve episodes with blood cultures obtained within 6 h of fever onset (N = 198 culture sets). Detailed cohort selection rationale is provided in Supplemental Methods.

### Statistical analysis

#### Time-to-antibiotics and clinical deterioration

We used logistic regression to estimate the association between TTA and the composite deterioration outcome. The primary analysis modeled TTA categorically (≤ 1 h vs > 1 h), reflecting the 1-h clinical threshold; secondary analyses modeled TTA continuously (per hour and log-transformed). We also compared three plausible correlates of antibiotic delay between TTA ≤ 1 h and TTA > 1 h episodes in the antibiotic-naïve cohort (fever-onset SOFA score, weekend versus weekday timing, and day shift [06:00–22:00] versus night shift timing) as a descriptive analysis of factors associated with timing variation (Supplemental Methods).

#### Bacteremia and mortality

We examined the association between recognized pathogen bacteremia and 90-day mortality in the antibiotic-naïve cohort (N = 224, primary), with the full cohort (N = 492) reported as a sensitivity. A broader any-positive culture definition was also analyzed as a sensitivity within each cohort.

#### Blood culture timing and yield

We compared culture positivity rates between cultures obtained before versus after antibiotic administration using logistic regression. At the episode level, we classified episodes by whether the first culture was drawn before or after empiric GN antibiotics. At the culture level, we examined all individual blood culture sets and modeled antibiotic timing as a continuous variable (hours from culture to antibiotic administration).

#### Robustness analysis

We performed robustness checks on the null primary finding, including a retrospective minimum detectable effect size analysis, the reverse fragility index for the categorical comparison, Firth's penalized regression and exact methods (Fisher's exact and 10,000-iteration permutation) for small-sample bias, finer TTA increments (per 15-min and per 30-min) and a log transformation for functional form, a fluid-bolus expanded composite outcome at three thresholds (≥ 1, ≥ 2, ≥ 3 boluses), a cohort-inclusion threshold sweep across upper TTA bounds of 2 to 12 h (Supplemental Results; Supplemental Tables S5, S7, S9, S12), and a sensitivity analysis restricted to episodes qualifying by a single temperature ≥ 38.3 °C. In a post-hoc sensitivity analysis addressing whether the fever-onset definition could shift the exposure start point, we repeated the primary adjusted categorical and per-hour models in the subset of episodes that reached a single temperature ≥ 38.3 °C (n = 145), for which no sustained-fever criterion applies.

Methods for microbiologic characterization, power and reverse fragility analysis, small-sample sensitivity analyses (Firth's penalized regression and exact methods), and the fluid-bolus expanded composite outcome are detailed in Supplemental Methods. All regression models were adjusted for baseline SOFA score, transplant type, day post-transplant, and age-adjusted Charlson comorbidity score.Clustered standard errors accounted for correlation among multiple episodes from the same patient. Analyses were performed using Python 3.11 with statsmodels. Two-sided P values < 0.05 were considered statistically significant [26, 27].

The authors used a large language model (Claude, Anthropic) to assist with code review of statistical analysis scripts and with verification of internal consistency between reported figures, tables, and text. All study design, data analysis, and interpretation were performed by the authors, who reviewed all AI-assisted output and take full responsibility for the integrity and accuracy of the work.

## Results

### Study population

**Fig. 1:**
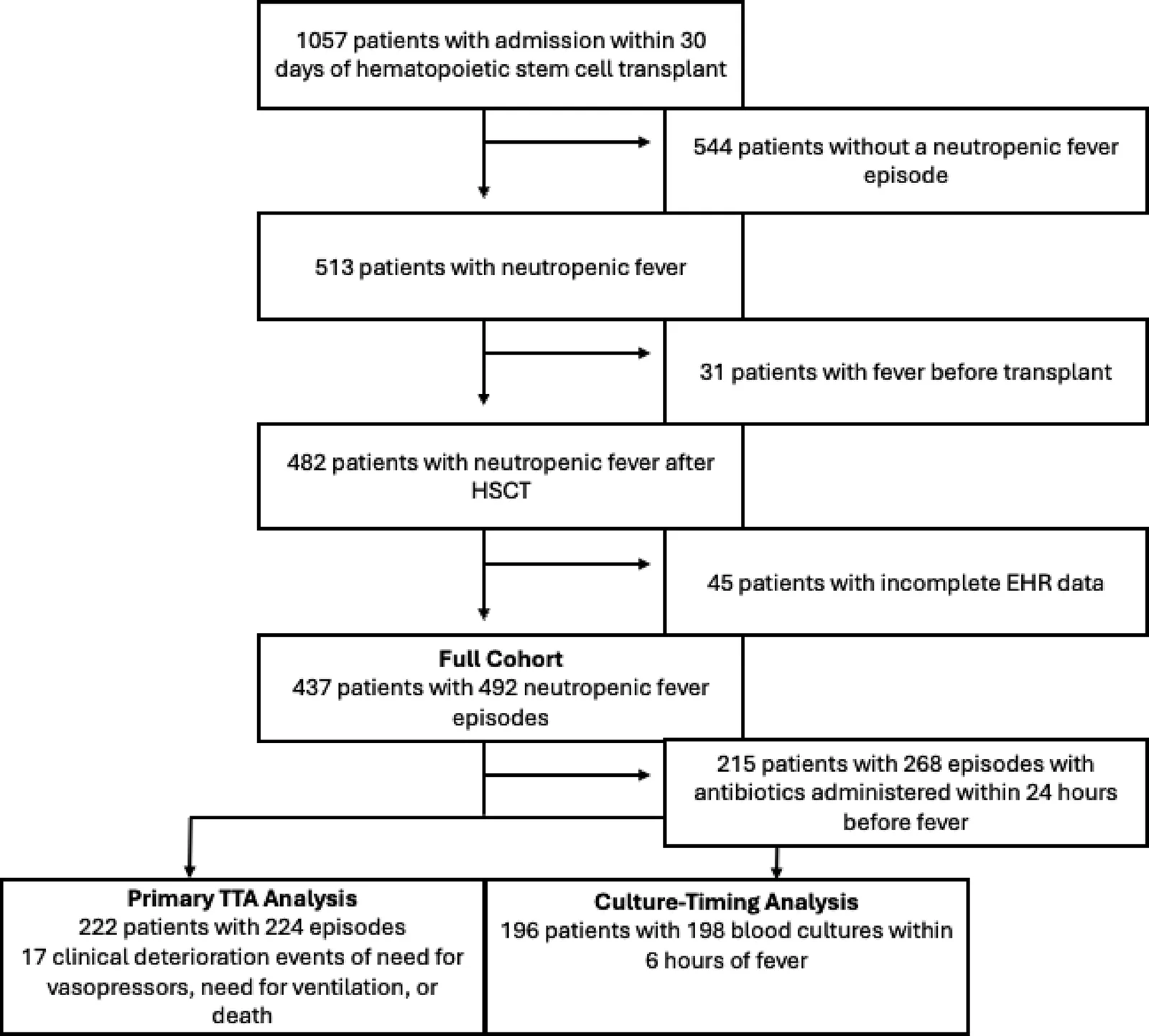
Study Cohort Flow Diagram

We identified 492 neutropenic fever episodes in 437 unique patients occurring within 30 days of HSCT. Cohort selection is summarized in Fig. 1 (CONSORT-style diagram). After restricting to antibiotic-naïve episodes with empiric gram-negative antibiotics administered within 12 h, 224 episodes remained for the primary time-to-antibiotics analysis. In this antibiotic-naïve cohort, the median age was 60 years (IQR 50–66), 96 (43%) were female, and 195 (87%) were White. The most common underlying diseases were myeloma (94, 42%), lymphoma (45, 20%), and acute myeloid leukemia (39, 17.4%). Sixty-eight episodes (30%) occurred in allogeneic transplant recipients (Table 1).

Neutropenic fever occurred at a median of 6.7 days (IQR 3.9–8.6) after transplantation. The median SOFA score at fever onset was 4 (IQR 3–5). Blood cultures were obtained in 219 episodes (98%), of which 69 (31%) had positive cultures and 59 (26%) grew recognized pathogens. The median time to empiric gram-negative antibiotics was 1.2 h (IQR 0.8–2.2); 86 episodes (38%) received antibiotics within 1 h of fever onset.

### Clinical outcomes

In the antibiotic-naïve cohort (N = 224), the composite clinical deterioration outcome occurred in 17 episodes (7.6%), comprising vasopressor requirement ≥ 6 h in 7 (3.1%), mechanical ventilation in 1 (0.4%), and 90-day mortality in 11 (4.9%). Notably, 10 of 11 deaths (91%) occurred without any preceding acute physiologic deterioration (i.e., no vasopressor requirement or mechanical ventilation) from the index neutropenic fever episode.

Detailed characterization of the 11 deaths (Supplemental Table S6, Supplemental Results) showed that 10 occurred without preceding sepsis trajectory at a median of 45 days after the index fever, with graft-versus-host disease, disease relapse, or progression of underlying disease as the dominant clinical context.

### Time-to-antibiotics and clinical deterioration

Among 224 antibiotic-naïve episodes with TTA ≤ 12 h, clinical deterioration occurred in 17 (7.6%). The distribution of TTA was right-skewed: 86 episodes (38.4%) received antibiotics within 1 h of fever onset, 160 (71.4%) within 2 h, 187 (83.5%) within 3 h, and 37 (16.5%) exceeded 3 h. In unadjusted analysis, deterioration occurred in 6 of 86 episodes (7.0%) with antibiotics administered ≤ 1 h versus 11 of 138 episodes (8.0%) with antibiotics > 1 h (odds ratio [OR] 0.87; 95% confidence interval [CI] 0.31–2.45). After adjusting for SOFA score, allogeneic transplant status, days from HSCT, and age-adjusted Charlson comorbidity score, receipt of antibiotics within 1 h was not associated with reduced deterioration (adjusted OR 1.18; 95% CI 0.37–3.78; P = 0.78) (Table 2).

**Table 2:** Association Between Time-to-Antibiotics and Clinical Deterioration

Model	Events/N	OR (95% CI)	P
***Primary analysis***			
TTA ≤ 1 h vs > 1 h (unadjusted)	6/86 vs 11/138	0.87 (0.31–2.45)	-
TTA ≤ 1 h vs > 1 h (adjusted)	17/224	1.18 (0.37–3.78)	0.78
***Secondary analyses (adjusted)***			
TTA per hour increase	17/224	1.03 (0.85–1.24)	0.80
TTA log-transformed	17/224	1.22 (0.66–2.25)	0.52

Adjusted models include SOFA score, allogeneic transplant status, days from HSCT, and age-adjusted Charlson comorbidity score. Standard errors clustered by patient. Clinical deterioration defined as vasopressor requirement ≥ 6 h, mechanical ventilation, or 90-day mortality

CI, confidence interval; HSCT, hematopoietic stem cell transplant; OR, odds ratio; SOFA, Sequential Organ Failure Assessment; TTA, time-to-antibiotics

Secondary analyses examining TTA as a continuous variable confirmed these findings. Each additional hour to antibiotic administration was not associated with deterioration (adjusted OR per hour 1.03; 95% CI 0.85–1.24; P = 0.80). Log-transformation of TTA yielded similar null results (OR 1.22; 95% CI 0.66–2.25; P = 0.52). Among covariates, only allogeneic transplant status was significantly associated with deterioration (adjusted OR 3.81; 95% CI 1.19–12.17; P = 0.02; full covariate coefficients in Supplemental Table S11).

### Robustness analyses

To quantify the study's sensitivity given the small number of events, we estimated the minimum detectable effect size: with 224 episodes and 17 deterioration events, the study had 80% power to detect a categorical odds ratio of 4.28 (≤ 1 h vs > 1 h) or a per-hour odds ratio of 1.41, and detecting a per-hour odds ratio of 1.20 would have required approximately 795 episodes (Supplemental Table S5). Sensitivity analyses across finer TTA increments (15-min, 30-min, per-hour), small-sample sensitivity analyses (Firth's penalized regression and exact methods), and an expanded composite outcome incorporating fluid bolus administration at three thresholds all supported the null primary finding (Supplemental Results; Supplemental Tables S5, S7, S9, S12). In a sensitivity analysis restricted to the 145 episodes that reached a single temperature ≥ 38.3 °C (13 deterioration events), the association remained null (TTA ≤ 1 h vs > 1 h, adjusted OR 1.39, 95% CI 0.35–5.51, P = 0.64; per hour, adjusted OR 1.04, 95% CI 0.70–1.54, P = 0.85). The reverse fragility index for the primary categorical comparison was 12, exceeding the 11 deterioration events observed in the > 1-h group. By 95% confidence interval, the upper bound on the per-hour TTA effect was OR 1.24.

### Bacteremia and 90-day mortality

In the antibiotic-naïve cohort (N = 224), recognized pathogen bacteremia occurred in 59 episodes (26.3%), with similar rates across underlying disease categories (Supplemental Table S4). Mortality at 90 days occurred in 4 of 59 recognized pathogen episodes (6.8%) versus 7 of 165 non-recognized pathogen episodes (4.2%). After adjustment for baseline SOFA score, transplant type, day post-transplant, and age-adjusted Charlson comorbidity score, recognized pathogen bacteremia was not associated with 90-day mortality (adjusted OR 1.98; 95% CI 0.50–7.90; P = 0.33) (Table 3). A sensitivity analysis using any-positive cultures (69/224, 30.8%) yielded similar findings (OR 1.15; 95% CI 0.31–4.28; P = 0.84). In the full cohort (N = 492), corresponding analyses were null (recognized pathogen 90/492, OR 0.85, 95% CI 0.35–2.08, P = 0.73; any-positive 111/492, OR 0.96, 95% CI 0.45–2.04, P = 0.92; Supplemental Table S8).

**Table 3:** Association Between Bacteremia and 90-Day Mortality

Bacteremia Definition	n/N (%)	Mortality (%)	Adj OR (95% CI)	P
Recognized pathogens	59/224 (26.3)	4/59 (6.8) vs 7/165 (4.2)	1.98 (0.50–7.90)	0.33
Any positive blood culture	69/224 (30.8)	4/69 (5.8) vs 7/155 (4.5)	1.15 (0.31–4.28)	0.84

Recognized pathogens excludes coagulase-negative staphylococci when present as a sole isolate and excludes fungal bloodstream infections. Adjusted for SOFA score, allogeneic transplant status, days from HSCT, and age-adjusted Charlson comorbidity score. Full-cohort (N = 492) sensitivity analyses are reported in Supplemental Table S8.

### Sensitivity analyses by outcome definition

We examined TTA associations across multiple outcome definitions in the antibiotic-naïve cohort. TTA was not significantly associated with any outcome tested (Table 4), strengthening confidence that the null finding is not an artifact of outcome misclassification.

**Table 4:** Sensitivity Analyses: TTA Associations by Outcome Definition

**Outcome**	**Events, N (%)**	TTA Adj OR/hour (95% CI)	**p**
Composite (primary)	17 (7.6)	1.03 (0.85–1.24)	0.80
Acute deterioration (shock or ventilation)	7 (3.1)	1.11 (0.87–1.42)	0.42
90-day mortality	11 (4.9)	0.93 (0.72–1.21)	0.60
Death without acute deterioration	10 (4.5)	0.95 (0.74–1.23)	0.70
Shock only (≥ 6 h vasopressors)	7 (3.1)	1.11 (0.87–1.42)	0.42

Per-hour TTA odds ratios across alternative outcome definitions. All analyses performed in the antibiotic-naïve cohort (N = 224). Adjusted for SOFA score, allogeneic transplant status, days from HSCT, and age-adjusted Charlson comorbidity score. Standard errors clustered by patient. The acute deterioration row and shock-only row are identical because no mechanical ventilation event occurred outside the context of shock in this cohort.

### Microbiology and empiric coverage

Among 69 bacteremic episodes in the antibiotic-naïve cohort, 89 isolates were identified (62.9% gram-negative, 37.1% gram-positive), with *Escherichia coli* (19), *Klebsiella pneumoniae* (15), coagulase-negative staphylococci (16), and *Pseudomonas aeruginosa* (9) as the most common organisms (Table 5). Among non-CoNS isolates, autologous recipients had predominantly gram-negative bacteremia (44 of 54, 81.5%) compared to allogeneic recipients (12 of 19, 63.2%), consistent with conditioning-induced mucosal-barrier translocation in autologous recipients (Supplemental Table S10). All 59 recognized pathogen bacteremic episodes received at least one empirically active antibiotic; no episode required regimen substitution. Multidrug resistance was rare (1 of 49 episodes with comprehensive AST after excluding intrinsic AmpC β-lactamase resistance; Supplemental Table S1) [28]. Oral mucositis of CTCAE grade ≥ 2 was documented in 61 of 224 episodes (27.2%). Detailed AMR profile (Supplemental Table S1), source of infection (Supplemental Table S2), and mucositis grades (Supplemental Table S3) are provided in Supplemental Results.

**Table 5:** Pathogen Distribution Among Bacteremic Episodes

Organism	Isolates, No
**Gram-Negative Organisms** (n = 56, 62.9%)	
*Escherichia coli*	19
*Klebsiella pneumoniae*	15
*Pseudomonas aeruginosa*	9
*Enterobacter cloacae* complex	5
*Klebsiella variicola*	5
*Klebsiella oxytoca*	1
*Fusobacterium* species	1
*Leptotrichia* species	1
**Gram-Positive Organisms** (n = 33, 37.1%)	
*Staphylococcus epidermidis*^a^	13
*Streptococcus salivarius* group	5
*Streptococcus mitis* group	4
*Staphylococcus aureus*	3
Coagulase-negative *Staphylococcus* species^a^	3
*Streptococcus bovis* group	1
*Streptococcus pneumoniae*	1
*Streptococcus canis* group	1
*Enterococcus faecium*	1
*Clostridium septicum*	1
**Total isolates**	**89**

Pathogen distribution among 69 bacteremic episodes in the antibiotic-naïve cohort (N = 224). Recognized bacterial pathogens included all organisms except coagulase-negative staphylococci when present as sole isolates and fungal isolates. Viridans group streptococci, *Leptotrichia* species, and other organisms sometimes considered contaminants in general medical populations were retained as recognized pathogens given their established pathogenicity in post-HSCT neutropenia. Coagulase-negative staphylococci treated as contamination when present as sole isolates per Methods.

### Antibiotic timing and blood culture positivity

Among 198 blood culture sets obtained within 6 h of fever onset in antibiotic-naïve episodes, 53 (26.8%) grew recognized bacterial pathogens. The majority (181/198, 91%) were obtained before empiric antibiotic administration. Culture positivity was similar in pre-antibiotic cultures (49/181, 27.1%) and post-antibiotic cultures (4/17, 23.5%); the difference was not statistically significant after adjustment (adjusted OR 1.03; 95% CI 0.28–3.85; P = 0.97).

## Discussion

In this retrospective cohort study of neutropenic fever episodes in HSCT recipients, we found no association between time-to-antibiotics and clinical deterioration among antibiotic-naïve patients, and no association between bacteremia and 90-day mortality. Strikingly, 10 of 11 deaths (91%) occurred without any preceding hemodynamic instability or respiratory failure, suggesting that mortality in this population is driven by underlying disease progression rather than the index febrile episode and subsequent sepsis or septic shock. These findings challenge the assumption that timing thresholds derived from septic shock cohorts should uniformly apply to post-transplant neutropenic fever.

The 1-h antibiotic standard for inpatient febrile neutropenia has been operationalized at major adult cancer centers without dedicated outcome evidence at that specific threshold. The 2010 IDSA guideline endorsed prompt empiric therapy but did not specify a 60-min target for adult inpatients; the explicit 1-h standard originates in the 2018 ASCO–IDSA outpatient management update, framed for emergency department and ambulatory presentation.1,4 The supporting outcome evidence traces primarily to septic shock cohorts in which delays in antimicrobial therapy from the onset of hypotension were associated with progressive mortality.9 Notably, the 2021 Surviving Sepsis Campaign guidelines themselves retreated from a strict 1-h threshold for sepsis without shock, recommending an extended 3-h window.10 Whether the 1-h target applies in adult inpatient HSCT recipients with neutropenic fever, a population that rarely presents with shock, has not been directly tested before the present analysis.

Our results are consistent with a growing body of evidence questioning the universal benefit of rapid antibiotics in febrile neutropenia outside the septic shock setting. A recent single-center analysis of adults with hematologic malignancies and febrile neutropenia found no association between hourly TTA and mortality and no signal at the ≤ 1-h, ≤ 2-h, or ≤ 3-h thresholds; an association emerged only after > 4 h and was attenuated in propensity-matched sepsis and bacteremia subgroups, paralleling our observation that bacteremia did not predict mortality.[29] In pediatric febrile neutropenia, an international individual patient data meta-analysis, a target trial emulation, and several recent observational studies have likewise failed to demonstrate a mortality benefit for TTA ≤ 1 h.18,[30–33] The 2020 systematic review by Koenig and colleagues found substantial heterogeneity across 13 studies, with a pooled mortality odds ratio of 0.78 (95% CI 0.16–3.69) failing to demonstrate consistent benefit.[12] Our findings extend this literature to the specific context of early post-transplant adult neutropenic fever, where patients are under continuous inpatient monitoring and empiric therapy is initiated rapidly regardless of minor timing variation.

That nearly all deaths occurred without preceding acute physiologic deterioration warrants emphasis. In sepsis cohorts presenting to the emergency department, death typically follows a trajectory of progressive organ dysfunction.[9, 10] In our cohort, this pattern was absent: only one of eleven deaths was preceded by shock or ventilation. The remaining ten are more plausibly attributed to relapse, graft-versus-host disease, or other complications of underlying disease than to the index febrile episode itself.

The pathophysiology of fever in early post-transplant neutropenia may explain these findings. Conditioning regimen toxicity causes mucosal barrier injury throughout the gastrointestinal tract, leading to translocation of enteric organisms that triggers fever and bacteremia without the fulminant course of community-acquired sepsis.19–21 The organism distribution in our cohort supports this mechanism: the predominant pathogens were enteric gram-negative rods (Escherichia coli, Klebsiella species, Enterobacter cloacae complex) and oral viridans group streptococci, organisms consistent with mucosal translocation rather than healthcare-associated infection. Notably, fluoroquinolone prophylaxis was used in only 5% of episodes, which likely contributed to the high bacteremia rate (31%) and the predominance of susceptible enteric organisms. In this context, the empiric antibiotic is functioning as treatment of bacterial translocation rather than as rescue therapy for deep-seated infection, so these patients may not face the same risk of decompensating to sepsis that the septic shock cohorts behind the 1-h standard presuppose.

Stratification by transplant type showed that the auto-predominant enteric pattern (81.5% non-CoNS gram-negative vs 63.2% in allogeneic recipients), likely reflecting severe mucositis from high-dose conditioning regimens such as melphalan, did not produce a detectable TTA effect (allogeneic adjusted OR 0.92 per hour, 95% CI 0.68–1.23, P = 0.57; autologous adjusted OR 1.22, 95% CI 0.95–1.56, P = 0.12; Supplemental Table S10). The absence of a TTA effect is therefore not driven by the pathophysiology of either transplant type.The lack of association between bacteremia and mortality deserves particular attention. Recognized pathogen bacteremia occurred in 26% of episodes, yet mortality rates were similar between bacteremic (4 of 59, 6.8%) and non-bacteremic (7 of 165, 4.2%) patients. This finding was robust to a sensitivity analysis using a broader any-positive culture definition and to analyses in the full cohort (Supplemental Table S8).While we cannot exclude residual confounding, this pattern is consistent with the hypothesis that early post-transplant bacteremia often represents transient, self-limited translocation events that resolve with standard empiric therapy regardless of precise timing.

Reasons for antibiotic delays in individual episodes were not consistently coded in structured EHR fields. As an indirect assessment, we examined three plausible correlates of delay in the antibiotic-naïve cohort. Fever-onset SOFA score was identical between TTA ≤ 1 h and > 1 h episodes (median 4.0, IQR 3.0–5.0 in both, P = 0.90), arguing against clinician triage of lower-acuity presentations as the dominant mechanism. Weekend timing was not associated with delay (P = 0.59). Day-shift fevers (06:00–22:00) were modestly more likely to be delayed than night-shift fevers (66.5% vs 50.7%, P = 0.04), a pattern more consistent with competing daytime clinical workflow (radiology, dialysis, scheduled antibiotic windows) than with failures of clinical recognition. The interpretation that operational delays do not adversely affect outcomes in this cohort assumes such delays predominantly reflect workflow variation rather than diagnostic uncertainty; future prospective work could resolve this distinction. Several limitations warrant consideration. First, our cohort was predominantly lower-risk: 156 of 224 episodes (69.6%) occurred in autologous transplant recipients, with a low median age-adjusted Charlson comorbidity score (1) and low 90-day mortality (4.9%). This composition, together with single-center prophylaxis and antimicrobial practices, limits generalizability to higher-risk populations, including centers with predominantly allogeneic transplantation, haploidentical or mismatched donors, myeloablative conditioning for high-risk disease, or a higher prevalence of multidrug-resistant organisms, in whom the consequences of antibiotic timing may differ. Second, the limited number of outcome events (17 deteriorations, 11 deaths) constrains statistical power and precluded organism-stratified analyses. Third, unmeasured confounding remains possible. Finally, the observational design cannot establish causation, and our results should be interpreted as hypothesis-generating. These limitations motivate our planned multicenter validation using national EHR data through COSMOS, which provides the scale needed to determine whether the null TTA finding generalizes beyond our single-center cohort.

These findings have implications for institutional targets and clinical practice. The 1-h antibiotic standard, while appropriate for undifferentiated sepsis in emergency settings, may not be an appropriate target for inpatient HSCT units where patients are continuously monitored and empiric therapy is reliably initiated within hours. Modest variation in timing (median 1.2 h, IQR 0.8–2.2) did not meaningfully influence outcomes in our cohort, potentially allowing clinicians additional time for clinical assessment, culture collection, and deliberate antibiotic selection. These findings should not be interpreted as supporting intentional delays in antibiotic administration, nor extrapolated to settings with prolonged treatment delays; patients presenting with hemodynamic instability should continue to receive antibiotics with maximum urgency per current best practice.

Among antibiotic-naïve HSCT recipients with neutropenic fever occurring within 30 days of transplantation, we found no significant associations between time-to-antibiotics and clinical deterioration or between bacteremia and mortality. The predominance of deaths without preceding acute deterioration suggests that mortality in this population reflects underlying disease rather than the index febrile episode. These hypothesis-generating findings support the need for multicenter studies to determine whether the 1-h standard, which originated for outpatient emergency management, should be uniformly applied to inpatient HSCT populations.

## Supplementary information

Below is the link to the electronic supplementary material.

Supplementary file1 (DOCX 37 KB)

Supplementary file2 (DOCX 22 KB)

## Data Availability

The data used in this study are not publicly available due to patient privacy and institutional policy.
