# Supplementary material for: Association between time-to-antibiotics and clinical outcomes in neutropenic fever after hematopoietic stem cell transplant
Source: Support Care Cancer. 2026 Oct 28;34(11):1162. doi: 10.1007/s00520-026-11141-8 (PMC13713999; doi:10.1007/s00520-026-11141-8)
Supplement: Supplementary file 1 [file 520_2026_11141_MOESM1_ESM.docx]

**Supplemental Tables**

**Supplemental Table S1.** Antimicrobial Resistance Patterns Among Bacteremic Isolates

**Supplemental Table S2.** Source of Infection Identification

**Supplemental Table S3.** Oral Mucositis Grades (CTCAE v5.0)

**Supplemental Table S4.** Bacteremia Rates by Underlying Disease

**Supplemental Table S5.** Statistical Robustness Analyses

**Supplemental Table S6.** Characterization of the 11 Deaths

**Supplemental Table S7.** Fluid-Bolus Expanded Composite Outcome Analysis

**Supplemental Table S8.** Bacteremia-Mortality Analysis: Primary (Antibiotic-Naïve N=224) and Sensitivity (Full N=492)

**Supplemental Table S9.** Time-to-Antibiotics at Finer Time Increments

**Supplemental Table S10.** Stratified Analyses by Transplant Type

**Supplemental Table S11.** Full Covariate Coefficients from Primary Regression Models

**Supplemental Table S12.** Cohort Inclusion Threshold Sweep

**Supplemental Table S1. Antimicrobial Resistance Patterns Among Bacteremic Isolates**

| **AST Data Availability** | **N (%)** |
| --- | --- |
| Bacteremic episodes (total) | 69 |
| Episodes with AST data | 61 (88.4%) |
| Without AST (external lab or not performed) | 8 (11.6%) |
| Total isolates with AST | 75 |
| Classifiable by Magiorakos framework | 56 (49 episodes) |
| Not covered (CoNS, streptococci) | 19 (18 episodes) |
| **Clinically Important Resistance Patterns** | **N (%)** |
| Methicillin-resistant Staphylococcus aureus (MRSA) | 0 |
| Extended-spectrum β-lactamase (ESBL) producers | 0 |
| Carbapenem-resistant Enterobacterales (CRE) | 0 |
| Vancomycin-resistant enterococci (VRE) bacteremia | 0 |
| Extensively drug-resistant (XDR) organisms | 0 |
| **MDR Classification** | **N/total (%)** |
| Strict Magiorakos (unified Enterobacteriaceae table) | 6/49 (12.2%) |
| After excluding intrinsic AmpC resistance in Enterobacter | 1/49 (2.0%) |

Magiorakos framework instructs exclusion of intrinsic resistance but provides a single Enterobacteriaceae table without species-level adjustment. Five of six MDR-flagged isolates were Enterobacter cloacae complex with resistance patterns consistent with intrinsic AmpC β-lactamase. The single clinically meaningful MDR isolate was an E. coli with acquired resistance to aminoglycosides, fluoroquinolones, and penicillin+BLI combinations.

**Supplemental Table S2. Source of Infection Identification**

| **Source** | **Positive / Tested** | **Notes** |
| --- | --- | --- |
| Bacteremia | 69/224 (30.8%) | Full organism data available (Table 5) |
| CT-confirmed pneumonia | 3/224 (1.3%) | 2 with Pseudomonas bacteremia, 1 non-bacteremic |
| Urinary tract infection (≥100k CFU) | 3/34 with urine cultures (8.8%) | All non-bacteremic; no urine-blood concordance |
| Clostridioides difficile (toxin-positive) | 7/146 tested (4.8%) | 6/7 non-bacteremic, independent entity |
| Respiratory viral panel positive | 3/50 panels (6.0%) | Rhinovirus/enterovirus (2 episodes), parainfluenza 3; all non-bacteremic |
| CMV viremia (ICD) | 1/224 (0.4%) | Non-bacteremic |
| **Total focal bacterial infection beyond bacteremia** | **6/224 (2.7%)** |  |

**Supplemental Table S3. Oral Mucositis Grades (CTCAE v5.0)**

| **CTCAE Grade** | **Description** | **N (%) of 224 episodes** |
| --- | --- | --- |
| None | No mucositis | 125 (55.8%) |
| Grade 1 | Asymptomatic or mild symptoms; intervention not indicated | 38 (17.0%) |
| Grade 2 | Moderate pain or ulcer that does not interfere with oral intake; modified diet indicated | 55 (24.6%) |
| Grade 3 | Severe pain; interfering with oral intake | 6 (2.7%) |
| Grade 4 | Life-threatening consequences; urgent intervention indicated | 0 (0%) |

Per-episode grade defined as the maximum recorded from 48 hours before fever onset through 24 hours after. Data capture from structured EHR nursing flowsheet assessments was 100%.

**Supplemental Table S4. Bacteremia Rates by Underlying Disease**

| **Underlying Disease** | **Bacteremic / Total** | **Rate (%)** |
| --- | --- | --- |
| Acute leukemia (AML + ALL) | 18/50 | 36.0 |
| Lymphoma | 11/45 | 24.4 |
| Multiple myeloma | 30/94 | 31.9 |
| Myelodysplastic syndromes | 0/1 | 0.0 |
| Other or unclassified | 10/34 | 29.4 |

Categories are mutually exclusive. Episodes were classified by primary diagnosis at HSCT, with myelodysplastic syndromes prioritized when also coded as acute leukemia. Two additional myelodysplastic syndrome episodes from the original cohort were excluded by the antibiotic-naive filter.

**Supplemental Table S5. Statistical Robustness Analyses**

| **Cohort Parameter** | **Value** |
| --- | --- |
| Antibiotic-naïve episodes | 224 |
| Clinical deterioration events | 17 |
| Event rate in ≤1-hour group | 6/86 (7.0%) |
| Event rate in >1-hour group | 11/138 (8.0%) |
| **Detectable Effects at 80% Power** | |
| Categorical OR (≤1 h vs >1 h) | ≥4.28 |
| Continuous per-hour OR | ≥1.41 |
| N required to detect per-hour OR 1.20 | ~795 |
| **Reverse Fragility** | |
| Additional events in >1 h group required for P<.05 | 12 |
| Events actually observed in >1 h group | 11 |
| **Effect Bound** | |
| Upper 95% CI on per-hour TTA effect | OR 1.24 |
| **Sensitivity Analyses** | |
| Categorical (≤1 h vs >1 h), Firth's penalized regression | OR 1.18 (0.42-3.28), P=.76 |
| Categorical (≤1 h vs >1 h), Fisher's exact | OR 1.16, P=1.00 |
| Per-hour, Firth's penalized regression | OR 1.04 (0.86-1.27), P=.68 |
| Per-hour, exact permutation (10,000 iter) | OR 1.03, P=.85 |

Statistical robustness analyses for the primary categorical TTA comparison (≤1 hour vs >1 hour). Detectable Effects at 80% power report the minimum effect sizes our analysis could detect given the observed 17 deterioration events; smaller per-hour effects (e.g., OR 1.20) would have required approximately 795 episodes. Reverse Fragility asks how many additional deterioration events in the >1-hour group would be required to push the comparison to statistical significance; this value (12) exceeds the events actually observed in that group (11), indicating robustness of the null finding to plausible event misclassification. Effect Bound reports the upper 95% confidence limit on the per-hour TTA effect from the observed data. Power computed via Hsieh's formula for logistic regression. Sensitivity Analyses report Firth's penalized regression (Heinze & Schemper, 2002), which provides bias-reduced estimates particularly informative under sparse outcomes, alongside exact methods (Fisher's exact for the categorical comparison; 10,000-iteration permutation test for the per-hour continuous comparison) that make no distributional assumptions. The fragility index does not establish equivalence but quantifies how readily additional events could shift the comparison.

**Supplemental Table S6. Characterization of the 11 Deaths**

| **Characteristic** | **N (%) of 11** |
| --- | --- |
| Allogeneic transplant | 8 (73) |
| Underlying acute myeloid leukemia | 6 (55) |
| Graft-versus-host disease documented | 3 (27) |
| Disease relapse documented | 2 (18) |
| Median days from index fever to death (range) | 45 (3-84) |
| Preceded by shock or ventilation (sepsis trajectory) | 1 (9) |
| Without preceding acute deterioration | 10 (91) |
| **Bacteremic at Index Fever (4 of 11 deaths)** | |
| Without intervening hemodynamic instability | 3 (75) |

The single death with preceding sepsis trajectory was an allogeneic AML recipient with Pseudomonas aeruginosa pneumonia and bacteremia at presentation; fever onset occurred on post-transplant day 11, with death 3 days later. The remaining 10 deaths occurred at a median of 45 days after the index fever, with graft-versus-host disease, disease relapse, late multi-organ dysfunction, or progression of underlying disease as the dominant clinical context.

**Supplemental Table S7. Fluid-Bolus Expanded Composite Outcome Analysis**

| **Composite Outcome Definition** | **Events / N (%)** | **Adjusted OR per Hour (95% CI)** | **p** |
| --- | --- | --- | --- |
| Original deterioration (vasopressors ≥6 h, ventilation, or 90-d death) | 17/224 (7.6) | 1.03 (0.85-1.24) | .80 |
| Original + ≥1 fluid bolus within 72 h | 136/224 (60.7) | 1.096 (0.936-1.284) | .254 |
| Original + ≥2 fluid boluses within 72 h | 94/224 (42.0) | 1.027 (0.892-1.183) | .706 |
| Original + ≥3 fluid boluses within 72 h | 58/224 (25.9) | 1.063 (0.925-1.220) | .390 |

Fluid bolus defined as single administration of ≥500mL of crystalloid or colloid. 131 of 224 episodes (58.5%) received at least one fluid bolus within the 72-hour window following fever onset. Adjusted for SOFA score, transplant type, days from HSCT, and age-adjusted Charlson comorbidity score. Standard errors clustered by patient.

**Supplemental Table S8. Bacteremia-Mortality Analysis: Primary (Antibiotic-Naïve N=224) and Sensitivity (Full N=492)**

| **Cohort** | **Analysis** | **Bacteremic / Total (%)** | **Deaths / Total (%)** | **Adjusted OR (95% CI)** | **p** |
| --- | --- | --- | --- | --- | --- |
| Antibiotic-naïve N=224 (primary) | Any positive bacteremia | 69/224 (30.8) | 11/224 (4.9) | 1.15 (0.31-4.28) | .84 |
| Antibiotic-naïve N=224 (primary) | Recognized pathogen | 59/224 (26.3) | 11/224 (4.9) | 1.98 (0.50-7.90) | .33 |
| Sensitivity (Full N=492) | Any positive bacteremia | 111/492 (22.6) | 47/492 (9.6) | 0.96 (0.45-2.04) | .92 |
| Sensitivity (Full N=492) | Recognized pathogen | 90/492 (18.3) | 47/492 (9.6) | 0.85 (0.35-2.08) | .73 |

The primary bacteremia-mortality analysis is restricted to the antibiotic-naïve N=224 cohort to match the time-to-antibiotic cohort. The regression was also re-run on the full N=492 cohort. The "recognized pathogen" analysis excludes isolates that did not represent a recognized clinically significant pathogen (coagulase-negative staphylococci as sole isolates, per the narrow CoNS definition). Both models adjusted for SOFA score, transplant type, days from HSCT, and age-adjusted Charlson comorbidity score; standard errors clustered by patient.

**Supplemental Table S9. Time-to-Antibiotics at Finer Time Increments**

| **TTA Parameterization** | **Adjusted OR (95% CI)** | **p** |
| --- | --- | --- |
| Per 15-minute increase | 1.006 (0.959-1.055) | .80 |
| Per 30-minute increase | 1.013 (0.921-1.114) | .80 |
| Per 1-hour increase (manuscript Table 2) | 1.026 (0.847-1.241) | .80 |
| Log-transformed (manuscript Table 2) | 1.22 (0.66-2.25) | .52 |

All models on the antibiotic-naïve cohort (N=224, 17 deterioration events). Adjusted for SOFA score, transplant type, days from HSCT, and age-adjusted Charlson comorbidity score. Standard errors clustered by patient. Finer time increments verify that the null per-hour finding is not an artifact of the per-hour parameterization.

**Supplemental Table S10. Stratified Analyses by Transplant Type**

|  | **Allogeneic (N=68)** | **Autologous (N=156)** |
| --- | --- | --- |
| **Clinical Outcomes (Antibiotic-Naïve Cohort)** | | |
| Any bacteremia, N (%) | 22 (32.4) | 47 (30.1) |
| Recognized-pathogen bacteremia, N (%) | 15 (22.1) | 44 (28.2) |
| Clinical deterioration, N (%) | 10 (14.7) | 7 (4.5) |
| 90-day mortality, N (%) | 8 (11.8) | 3 (1.9) |
| SOFA at fever onset, median (IQR) | 4 (3-5) | 4 (3-5) |
| Days from HSCT to NF, median (IQR) | 4.2 (1.8-8.5) | 6.8 (4.7-8.6) |
| **Organism Distribution (non-CoNS isolates within recognized-pathogen episodes)** | | |
| Gram-negative isolates, N (%) | 12 (63.2) | 44 (81.5) |
| Gram-positive isolates, N (%) | 7 (36.8) | 10 (18.5) |
| Non-CoNS isolates, total | 19 | 54 |
| **Stratified TTA → Deterioration** | | |
| N / events | 68 / 10 | 156 / 7 |
| TTA per hour, adjusted OR (95% CI) | 0.92 (0.68-1.23) | 1.22 (0.95-1.56) |
| P value | .57 | .12 |

Gram-stain counts are restricted to non-CoNS isolates within episodes containing at least one recognized pathogen, consistent with the recognized-pathogen analysis in Supp Table S8; ten episodes (7 allogeneic, 3 autologous) with coagulase-negative staphylococci as the sole isolate were excluded as probable contamination. Within-stratum regressions adjusted for SOFA score, age-adjusted Charlson comorbidity score, and days from HSCT; standard errors clustered by patient. The transplant covariate is constant within strata and was therefore omitted from the within-stratum models.

**Supplemental Table S11. Full Covariate Coefficients from Primary Regression Models**

| **Covariate** | **Adjusted OR (95% CI)** | **P** |
| --- | --- | --- |
| **Model 1: Categorical TTA (≤1 h vs >1 h)** | | |
| TTA ≤1 hour (vs >1 hour) | 1.18 (0.37-3.78) | .78 |
| SOFA score at fever onset | 1.08 (0.75-1.56) | .69 |
| Allogeneic transplant | 3.81 (1.19-12.17) | .02 |
| Age-adjusted Charlson comorbidity score | 1.01 (0.98-1.04) | .43 |
| Days from HSCT to NF onset | 0.98 (0.81-1.18) | .84 |
| **Model 2: Continuous TTA Per Hour** | | |
| TTA, per hour | 1.03 (0.85-1.24) | .80 |
| SOFA score at fever onset | 1.07 (0.74-1.54) | .71 |
| Allogeneic transplant | 3.63 (1.20-10.96) | .02 |
| Age-adjusted Charlson comorbidity score | 1.01 (0.98-1.04) | .43 |
| Days from HSCT to NF onset | 0.98 (0.82-1.18) | .85 |
| **Model 3: Log-Transformed TTA** | | |
| TTA, log-transformed | 1.22 (0.66-2.25) | .52 |
| SOFA score at fever onset | 1.06 (0.74-1.53) | .74 |
| Allogeneic transplant | 3.52 (1.15-10.79) | .03 |
| Age-adjusted Charlson comorbidity score | 1.01 (0.98-1.04) | .42 |
| Days from HSCT to NF onset | 0.98 (0.82-1.19) | .87 |

All three primary regression models on the antibiotic-naïve cohort (N=224, 17 deterioration events). Outcome: clinical deterioration (vasopressor requirement ≥6 h, mechanical ventilation, or 90-day mortality). Standard errors clustered by patient.

**Supplemental Table S12. Cohort Inclusion Threshold Sweep**

| **Threshold** | **N** | **Events** | **Categorical OR ≤1h vs >1h (95% CI)** | **P** | **Continuous OR per hour (95% CI)** | **P** |
| --- | --- | --- | --- | --- | --- | --- |
| TTA ≤ 2h | 160 | 12 | 1.44 (0.34-6.06) | .62 | 1.53 (0.35-6.67) | .57 |
| TTA ≤ 3h | 187 | 13 | 1.62 (0.42-6.22) | .48 | 1.02 (0.37-2.80) | .97 |
| TTA ≤ 4h | 200 | 13 | 1.80 (0.48-6.73) | .39 | 0.81 (0.38-1.72) | .58 |
| TTA ≤ 6h | 209 | 15 | 1.33 (0.39-4.45) | .65 | 1.25 (0.72-2.16) | .43 |
| TTA ≤ 12h (primary) | 224 | 17 | 1.18 (0.37-3.78) | .78 | 1.03 (0.85-1.24) | .80 |

For each threshold T, the antibiotic-naïve cohort was restricted to episodes with TTA ≤ T hours; the primary categorical analysis (TTA ≤1h vs >1h) and a continuous per-hour analysis were re-run within each restricted cohort. All models adjusted for SOFA score, allogeneic transplant status, age-adjusted Charlson comorbidity score, and days from HSCT; standard errors clustered by patient.
