# Supplementary material for: Association between time-to-antibiotics and clinical outcomes in neutropenic fever after hematopoietic stem cell transplant
Source: Support Care Cancer. 2026 Oct 28;34(11):1162. doi: 10.1007/s00520-026-11141-8 (PMC13713999; doi:10.1007/s00520-026-11141-8)
Supplement: Supplementary file 2 [file 520_2026_11141_MOESM2_ESM.docx]

# **Supplemental Methods** **Neutropenic Fever Definition Rationale** The sustained-fever criterion (≥38.0°C for ≥2 hours) reflects the more conservative of two commonly used definitions and was selected to reduce inclusion of transient elevations that do not prompt clinical action; in our EHR data this was operationalized as two recorded temperatures ≥38.0°C sustained over a 2-hour span, with episode onset assigned to the first qualifying temperature. The ANC threshold of <1500 cells/µL is broader than the IDSA febrile neutropenia threshold of <500 cells/µL and was selected to capture episodes occurring during the peri-engraftment transition, when ANC may fluctuate above and below 500 cells/µL between consecutive measurements.

## **Study Cohort Selection Rationale** For the primary time-to-antibiotics analysis, we restricted to antibiotic-naïve episodes in which empiric gram-negative antibiotics were administered within 12 hours of fever onset (N=224). TTA can only be meaningfully measured in patients who had not received intravenous antibiotics in the preceding 24 hours, and the 12-hour ceiling was selected to focus on clinically relevant variation in timing while excluding episodes with atypical treatment patterns. For culture-timing analyses, we examined antibiotic-naïve episodes with blood cultures obtained within 6 hours of fever onset (N=198 culture sets) because the pre-versus-post-empiric classification can only be made when both the culture timestamp and the empiric-antibiotic timestamp are known.

**Power and Robustness Analyses**
We conducted a retrospective power analysis under the observed cohort parameters (N=224 antibiotic-naïve episodes, 17 deterioration events) to determine the minimum detectable effect size at 80% power. We additionally computed the reverse fragility index for the primary categorical contrast (the number of additional events required in the >1-hour group to reach P<.05).31 We performed small-sample sensitivity analyses using Firth's penalized regression and exact methods (Fisher's exact for the categorical comparison; 10,000-iteration permutation for the per-hour comparison) to address potential small-sample bias in maximum-likelihood estimation.32 We additionally performed sensitivity analyses examining bacteremia and TTA associations across alternative outcome definitions including acute deterioration alone (shock or ventilation without mortality), mortality alone, and shock alone.

**Cohort-Inclusion Threshold Sensitivity**
To assess sensitivity of the null finding to the 12-hour cohort-inclusion ceiling used in the primary analysis, the categorical (≤1h vs >1h) and continuous per-hour analyses were re-run within progressively narrower antibiotic-naïve subcohorts defined by TTA upper bounds of 2, 3, 4, 6, and 12 hours (Supplemental Table S12). Each restricted cohort used the same four-covariate adjustment (SOFA, allogeneic transplant, days from HSCT, age-adjusted Charlson) and patient-level clustering as the primary analysis.

**Correlates of Antibiotic Delay**
To provide indirect context for variation in time-to-antibiotics, three plausible correlates of delay were compared between TTA ≤1h and TTA >1h episodes in the antibiotic-naïve cohort (N=224): fever-onset SOFA score, weekend versus weekday fever onset, and day shift (06:00–22:00) versus night shift fever onset. Continuous variables were compared with Wilcoxon rank-sum tests and binary classifications with Fisher's exact tests.

**Fluid-Bolus Composite Outcome**
As a secondary sensitivity analysis on the deterioration definition, we constructed an expanded composite that added intravenous fluid bolus administration to the original components of the deterioration outcome. Bolus events were identified from the medication administration record using validated drug-name and volume filters (≥500 mL of crystalloid or colloid fluids). Three bolus thresholds were tested (≥1, ≥2, ≥3 boluses within the 72-hour window following fever onset).

**Microbiologic Characterization**
For all bacteremic episodes, organism identity and gram classification were extracted from the structured microbiology record (or recovered from primary microbiology data for the 5 episodes whose susceptibility testing had been sent to an external reference laboratory or had not been completed). Empiric coverage adequacy was assessed by crosswalking each empiric antibiotic given within 12 hours of fever onset against the susceptibility profile of the isolated organism. For episodes with institutional susceptibility panels, this was a direct AST lookup; for episodes recovered from primary microbiology, coverage was verified against published susceptibility data for the recovered organism. Multidrug resistance was classified per the Magiorakos consensus criteria using institutional AST data.33

**Source of Infection**
Non-bacteremic infectious sources were extracted from the structured EHR within the 7-day fever window. Focal bacterial infections comprised CT-confirmed pneumonia (manual review of chest CT radiology reports for explicit pneumonia findings), urinary tract infection (urine culture ≥10⁵ CFU/mL of a recognized urinary pathogen; denominator restricted to episodes with urine culture results), and tracheal aspirate bacterial culture growth. Clostridioides difficile infection required a stool toxin-positive result (denominator restricted to episodes with stool testing). Respiratory viral co-infection was identified from respiratory viral panel results (denominator restricted to episodes with viral testing). Cytomegalovirus viremia was identified from CMV PCR positivity within the fever window.

**Mucositis Grading**
Oral mucositis grade was extracted from the structured CTCAE v5.0 assessment recorded in the EHR within the 72-hour window surrounding fever onset.

**Supplemental Results** **Detailed Characterization of the 11 Deaths**
Detailed characterization of the 11 deaths (Supplemental Table S6) showed that 8 (73%) occurred in allogeneic transplant recipients (compared with 30% of the cohort overall), 6 (55%) had underlying acute myeloid leukemia, and the median interval from index fever to death was 45 days (range 3-84). Graft-versus-host disease was documented in 3 (27%) and disease relapse in 2 (18%). Only one death, an allogeneic AML recipient with Pseudomonas aeruginosa pneumonia and bacteremia at presentation (fever onset on post-transplant day 11; death three days later), was preceded by acute hemodynamic and respiratory deterioration attributable to the index febrile episode. The remaining 10 deaths occurred without preceding sepsis trajectory, at a median of 45 days after the index fever, with graft-versus-host disease, disease relapse, late multi-organ dysfunction, or progression of underlying disease as the dominant clinical context. Among the 4 deaths occurring in patients bacteremic at the index fever, 3 (75%) occurred without intervening hemodynamic instability, a pattern more consistent with transient mucosal-barrier translocation that resolved than with progressive bacterial sepsis from the index episode.

**Robustness analyses** **Power and Reverse Fragility**
With 224 antibiotic-naïve episodes and 17 deterioration events, our study had approximately 80% power to detect a categorical odds ratio of 4.28 or greater (≤1 hour vs >1 hour) and a per-hour odds ratio of approximately 1.41 or greater for the continuous TTA model; detecting smaller per-hour effects (e.g., OR 1.20) would have required approximately 795 episodes (Supplemental Table S5). The reverse fragility index for the primary categorical contrast was 12: twelve additional deterioration events in the >1-hour group would be required to push the comparison to statistical significance, a value exceeding the eleven deterioration events actually observed in that group. While these analyses do not establish equivalence, they meaningfully constrain the upper bound of the TTA effect (per-hour OR ≤1.24 by 95% confidence interval). Small-sample sensitivity analyses returned results consistent with the primary analysis: Firth's penalized regression gave categorical OR 1.18 (P=.76) and per-hour OR 1.04 (P=.68); exact methods (Fisher's exact, 10,000-iteration permutation) returned P=1.00 and P=.85, respectively (Supplemental Table S5).

**Cohort-Inclusion Threshold Sensitivity**
The categorical TTA association remained nonsignificant across all cohort inclusion thresholds: TTA ≤2h categorical OR 1.44 (95% CI 0.34-6.06); TTA ≤3h OR 1.62 (0.42-6.22); TTA ≤4h OR 1.80 (0.48-6.73); TTA ≤6h OR 1.33 (0.39-4.45); TTA ≤12h (primary) OR 1.18 (0.37-3.78). Continuous per-hour estimates were similarly null across all thresholds (range 0.81 to 1.53, all P>.43; Supplemental Table S12). The conclusion that TTA was not associated with deterioration was stable across all inclusion thresholds examined.

**Functional Form of TTA Exposure**
Sensitivity analyses modeling TTA at finer time increments returned consistent null associations: per 15-minute OR 1.006 (95% CI 0.959-1.055), P=.80; per 30-minute OR 1.013 (0.921-1.114), P=.80; per 1-hour OR 1.026 (0.847-1.241), P=.80 (Supplemental Table S9). We considered categorical analyses at 2-hour and 3-hour thresholds but did not pursue them given that only 5 deterioration events occurred in the >2-hour group and 4 in the >3-hour group, rendering such comparisons severely underpowered.

### **Fluid-Bolus Composite Outcome** As a sensitivity analysis on the deterioration definition, we constructed expanded composite outcomes adding intravenous fluid bolus administration at three thresholds (Supplemental Table S7). One hundred thirty-one of 224 episodes (58.5%) received at least one fluid bolus within the relevant window. Each expanded outcome remained null: ≥1 bolus composite 136/224 events (60.7%), adjusted OR per hour 1.096 (95% CI 0.936-1.284), P=.254; ≥2 boluses 94/224 (42.0%), OR 1.027 (0.892-1.183), P=.706; ≥3 boluses 58/224 (25.9%), OR 1.063 (0.925-1.220), P=.390.

## **Microbiology and Empiric Coverage** **Pathogen Distribution** Among the 69 bacteremic episodes in the antibiotic-naïve cohort, 89 isolates were identified: 56 (62.9%) gram-negative and 33 (37.1%) gram-positive (Table 5). The most common organisms were Escherichia coli (19), Klebsiella pneumoniae (15), coagulase-negative staphylococci (16), and Pseudomonas aeruginosa (9). Among non-CoNS isolates, allogeneic recipients (N=68) had 12 (63.2%) gram-negative and 7 (36.8%) gram-positive; autologous recipients (N=156) had 44 (81.5%) gram-negative and 10 (18.5%) gram-positive (Supplemental Table S10). The higher proportion of enteric gram-negative organisms in autologous recipients likely reflects severe GI mucositis from high-dose conditioning regimens such as melphalan.

### **Antimicrobial Resistance** Of 49 episodes with comprehensive institutional susceptibility data, 1 (2.0%) met Magiorakos consensus criteria for acquired multidrug resistance after excluding intrinsic AmpC β-lactamase resistance in Enterobacter cloacae complex isolates (Supplemental Table S1). We identified no extended-spectrum β-lactamase producers, carbapenem-resistant Enterobacterales, methicillin-resistant Staphylococcus aureus, vancomycin-resistant enterococci, or extensively drug-resistant organisms.

### **Empiric Coverage Adequacy** Of the 69 bacteremic episodes in the antibiotic-naïve cohort, 59 grew a recognized pathogen (10 episodes with coagulase-negative staphylococci as sole isolates were classified as contamination per Methods). All 59 (100%) received at least one empiric antibiotic active against the eventually-identified pathogen. Coverage adequacy was assessed using institutional susceptibility panels where available; for the five episodes whose susceptibility testing had been sent to an external reference laboratory, organism identity was recovered from primary microbiology data and coverage verified against published susceptibility data. The single AST panel limitation was one Streptococcus canis bacteremia for which cefepime was not on the susceptibility panel; S. canis is universally cefepime-susceptible. No episode required regimen extension or substitution based on susceptibility results.

### **Source of Infection** Focal bacterial infections beyond bacteremia were identified in 6 of 224 episodes (2.7%): CT-confirmed pneumonia in 3 (1.3%) and urinary tract infection (≥10⁵ CFU/mL) in 3 of 34 episodes with urine cultures (8.8%) (Supplemental Table S2). Clostridioides difficile infection (toxin-positive) was identified in 7 of 146 episodes with stool testing (4.8%). Respiratory viral co-infection was identified in 3 of 50 episodes with viral panel testing (6.0%). Cytomegalovirus viremia was identified in 1 of 224 episodes (0.4%).

### **Mucositis** Grade ≥2 mucositis per CTCAE v5.0 criteria was documented in 61 of 224 episodes (27.2%), with 100% data capture from structured EHR assessments (Supplemental Table S3).
